# Optimizing Cabozantinib Dosing in Unresectable Hepatocellular Carcinoma of 7-on/7-off Regimen

**DOI:** 10.3390/cancers17081288

**Published:** 2025-04-10

**Authors:** Yudai Fujiwara, Hidekatsu Kuroda, Tamami Abe, Kei Endo, Takayoshi Oikawa, Mio Tsuruoka, Masashi Ninomiya, Masashi Fujita, Kazumichi Abe, Tomohiro Katsumi, Shinichiro Minami, Wataru Sato, Go Igarashi, Chikara Iino, Nobukazu Tanabe, Hiroshi Numao, Osamu Kimura, Ippeki Nakaya, Asami Ito, Takuya Watanabe, Kenji Yusa, Tomoaki Nagasawa, Hiroki Sato, Akiko Suzuki, Yuichi Yoshida, Kei Sawara, Keisuke Kakisaka, Akio Miyasaka, Hiromasa Ohira, Yoshiyuki Ueno, Takayuki Matsumoto

**Affiliations:** 1 Division of Gastroenterology and Hepatology, Department of Internal Medicine, Iwate Medical University, Iwate 0283695, Japan; 2 Division of Gastroenterology, Tohoku University Graduate School of Medicine, Miyagi 9808574, Japan; 3 Department of Gastroenterology, Fukushima Medical University School of Medicine, Fukushima 96012950, Japan; 4 Department of Gastroenterology, Faculty of Medicine, Yamagata University, Yamagata 9902311, Japan; 5 Department of Gastroenterology, Graduate School of Medicine, Akita University, Akita 0100041, Japan; 6 Department of Gastroenterology, Hirosaki University Graduate School of Medicine, Aomori 0368563, Japan; 7 Department of Gastroenterology, National Hospital Organization Sendai Medical Center, Miyagi 9830045, Japan; 8 Department of Gastroenterology, Aomori Prefectural Central Hospital, Aomori 0300913, Japan; 9 Department of Gastroenterology, South Miyagi Medical Center, Miyagi 9891253, Japan

**Keywords:** cabozantinib, hepatocellular carcinoma, adverse events, tyrosine kinase inhibitor, ALBI score

## Abstract

Cabozantinib (CAB) causes a high incidence of adverse events in unresectable hepatocellular carcinoma (u-HCC) and requires dose adjustments. We evaluated the efficacy and safety of the 7-on/7-off regimen composed of 7 consecutive days’ administration of CAB followed by a 7-day rest period. This was a retrospective analysis of 35 patients with u-HCC, who were treated with CAB in a multicenter cohort in Japan from 2020 to 2024. The clinical outcomes of 12 patients treated with the 7-on/7-off regimen and 23 patients treated with daily dosing were compared. There were significant differences in overall survival (12.4 months vs. 6.3 months, *p* = 0.03), median progression-free survival (4.8 months vs. 3.2 months, *p* < 0.01), objective response rate (16.7% vs. 0.0%, *p* = 0.04), and incidence of any adverse events (75.0% vs. 100.0%, *p* = 0.03) between the 7-on/7-off regimen group and daily dosing group. The median duration of drug exposure (122 days vs. 42 days, *p* < 0.01) and median duration of dose reduction (100 days vs. 23 days, *p* < 0.01) were prolonged significantly in the 7-on/7-off group than in the daily dosing group. CAB in a 7-on/7-off regimen may improve the tolerability and treatment response in patients with u-HCC.

## Simple Summary

Administration of cabozantinib for unresectable hepatocellular carcinoma frequently requires dose reduction owing to adverse events. Therefore, we developed and evaluated the efficacy and safety of a regimen of alternating 7 days of cabozantinib administration with 7 days of rest. The intermittent dosing group demonstrated prolonged overall survival and progression-free survival in comparison to the daily dosing group, along with a reduced incidence of early adverse events. Furthermore, the intermittent dosing group experienced extended drug exposure and delayed time to dose reduction. These findings suggest that the 7-day-on/7-day-off cabozantinib regimen may significantly improve both tolerability and treatment response in patients with unresectable hepatocellular carcinoma, potentially offering a more effective approach to managing this challenging disease.

## 1. Introduction

Hepatocellular carcinoma (HCC) is the sixth most common cancer worldwide, and its poor prognosis makes it the third leading cause of cancer-related death [1]. As a systemic therapy for unresectable HCC (u-HCC), cabozantinib (CAB) is considered a second-line treatment option after atezolizumab plus bevacizumab or durvalumab plus tremelimumab [2,3].

CAB is a tyrosine kinase inhibitor (TKI), which exerts its activity primarily by inhibiting vascular endothelial growth factor receptor (VEGF-) 2. It also inhibits other tyrosine kinases, such as MET, AXL, RET, KIT, FLT3, and TYRO [4]. In the CELESTIAL trial, the median overall survival (OS) was 10.2 months in the CAB-treated group and 8.0 months in the placebo group (hazard ratio 0.76, 95% confidence interval 0.63–0.92, *p* = 0.005), with a significant survival benefit in the form. Consequently, CAB has been available for the treatment of u-HCC since 2020 in Japan [5]. It is noteworthy that CAB had the required efficacy despite the need for dose reduction in many of the study participants. The initial dose of CAB for u-HCC is 60 mg once daily, but 62% of patients in the CELESTIAL study [5] and 91% of patients in the Japanese phase II study [6] had treatment-related adverse events (AEs), which required dose reduction. Therefore, the efficacy and safety of the optimized initial dose of CAB to 40 or 20 mg/day have been examined [7,8].

The plasma elimination half-life of CAB is 120 h [9], the duration of which is longer than that of other TKIs such as lenvatinib (27 h) [10], regorafenib (28 h) [11], and sorafenib (20–48 h) [12]. In a study investigating an exposure–response analysis of CAB for metastatic renal cell carcinoma, steady-state plasma CAB trough concentration (C_trough_) was low with average values of 465.6 ng/mL in patients with progressive disease, and it was high with a value of 950.2 ng/mL in patients with grade 3 or greater AEs [13]. Similarly, an exposure–response analysis of CAB for u-HCC showed benefits with greater OS and progression-free survival (PFS) in 60 or 40 mg/day CAB than in 20 mg/day [14]. In contrast, the incidence of AEs was lower in 20 or 40 mg/day CAB than in 60 mg/day. These observations suggest a need for revising drug protocols to balance the therapeutic effect and AEs by optimizing the dosing schedule. However, there have been few reports of the daily administration of reduced-dose CAB. Furthermore, there have been no attempts to evaluate the efficacy and tolerability of on-and-off dosing protocols in consideration of the long half-life of the drug.

We therefore proposed a “7-on/7-off” regimen of CAB with a cyclic dosing schedule comprising 7-day consecutive administration of 40 mg/day followed by a 7-day rest period. In this study, we analyzed real-world clinical outcomes from multiple Japanese institutions and evaluated the therapeutic efficacy and tolerability of the regimen in patients with u-HCC.

## 2. Materials and Methods

### 2.1. Patients

This was a retrospective, multicenter cohort study conducted in Japanese institutions majoring in HCC. Patients were recruited from nine liver centers (Iwate Medical University Hospital, Hirosaki University Hospital, Aomori Prefectural Central Hospital, Akita University Hospital, Tohoku University Hospital, National Hospital Organization Sendai Medical Center, South Miyagi Medical Center, Yamagata University Hospital, and Fukushima Medical University Hospital). We enrolled 35 patients with intermediate or advanced-stage HCC, who were treated with CAB from November 2020 until December 2024 (total study duration of 4 years and 1 month). The inclusion criteria for study registration were as follows: (i) A diagnosis of u-HCC based on findings from a tumor-targeted biopsy or imaging evaluation; (ii) Measurable lesions depicted on computed tomography (CT) [15] and magnetic resonance imaging (MRI) [16,17]; (iii) A Child–Pugh Score of 5–7 [18]; (iv) BCLC stage B or C [19]; (v) An Eastern Cooperative Oncology Group (ECOG) performance status score of 0 or 1 [20]. Patients were classified into two groups according to the applied protocols of CAB, either the daily dosing group or 7-on/7-off group.

The study protocol of this study was approved by the institutional review board of Iwate Medical University (approval number: MH 2020-155). The patients gave their written informed consent before treatment in accordance with the ethical standards of the 2013 Declaration of Helsinki (Fortaleza revision, 2013) and its subsequent amendments.

### 2.2. Simulation of Plasma Concentration

According to the exposure–response analysis of CAB [13] conducted by Cerbone et al., C_trough_ values ranging from 470 to 950 ng/mL were applied to ensure drug efficacy and safety in this study. The median C_max_ for CAB (40 mg) was 251 ng/mL, with an area under the concentration–time curve for 24 h of 4158 ng·h/mL and a time to maximum concentration of 4 h [21]. The half-life of the terminal elimination phase was established at 120 h [9]. We simulated the blood concentration of the drug for the daily dosing and 7-on/7-off regimen of CAB using a two-compartment model.

### 2.3. Treatment Protocol

The decision-making for dosing schedules of CAB was left to the discretion of each institution. For patients in the daily dosing group, the recommended initial dose of CAB was 60 mg. However, the initial dose was adjusted as 40 mg or 20 mg according to the decision by the attending physician. In the early phase of this study, we had more experience with daily dosing than with the 7-on/7-off regimen. Consequently, the initial dose at 20 or 40 mg was chosen for all patients treated by the 7-on/7-off regimen. The chemotherapy cycle of CAB administration was defined as 28 days.

According to the CAB administration guidelines [5], the drug dose was adjusted or discontinued for patients experiencing severe AEs (grade ≥ 3) or any unacceptable grade 2 drug-related AEs. AEs were evaluated using version 6.0 of the National Cancer Institute Common Terminology Criteria for Adverse Events [21]. The dose reduction of CAB was carried out at the discretion of each facility. CAB therapy continued until disease progression, unacceptable toxicity, or withdrawal of consent. Relative dose intensity (RDI) was calculated by dividing the actual dose by the ideal dose for the entire CAB period.

### 2.4. Assessments of Hepatic Functional Reserve and Therapeutic Response

The Child–Pugh classification [18] and modified albumin-bilirubin (mALBI) grade [22] were employed to assess liver function. The mALBI score was calculated both when CAB was initiated and at the end of treatment.

Dynamic CT or MRI was used to assess the therapeutic response to CAB according to the Response Evaluation Criteria in Solid Tumors (RECIST) version 1.1 [23] and the modified RECIST (mRECIST) [24]. Tumors were assessed once during the initial eight weeks and subsequently at the discretion of each facility. The objective response rate (ORR) was defined as the percentage of patients with a complete (CR) or a partial (PR) response. The disease control rate (DCR) was defined as the percentage of patients with a CR, PR, or stable disease (SD). OS was defined as the time interval from the initiation of CAB treatment to death from any cause. PFS was defined as the duration from the first administration of CAB to either disease progression (assessed according to mRECIST) or death.

### 2.5. Statistical Analysis

All statistical analyses were conducted using EZR (Saitama Medical Center, Jichi Medical University, Saitama, Japan), a modified version of R commander that adds statistical functions commonly used in biostatistics, in conjunction with R (the R foundation for statistical computing, Vienna, Austria) [25].

Continuous variables were presented as the median (interquartile range) and analyzed using either Student’s *t*-test or the Mann–Whitney U test. Categorical variables are expressed as the number (percentage) and analyzed using either Pearson’s chi-square test or Fisher’s exact test. The Kaplan–Meier method was used to create OS and PFS curves, which were compared using the log-rank test. A mixed-effects regression model with random intercepts was employed to determine if there was a difference in the rate of change in mALBI between 7-on/7-off and daily dosing groups. This was assessed by comparing the slope of changes in the mALBI score between the start and the end of treatment. *p* values of <0.05 were considered to indicate statistical significance.

## 3. Results

### 3.1. Patient Characteristics

Background demographics and characteristics of the subjects are summarized in Table 1. The median age was 67.2 years, and 32 patients (91.4%) were male. CAB was given as the second-line therapy in 15 patients and as the third- or later-line therapy in 20 patients. mALBI grade 1 was noted in 15 patients. Eighty percent (28 patients) had BCLC stage C disease. The median tumor size was 6.9 cm. A total of 82.9% had multiple tumors, 13 (44.8%) had macrovascular invasion, and 18 (62.1%) had extrahepatic spread. There were no significant differences in baseline factors between the 7-on/7-off and the daily dosing groups.

### 3.2. Simulation of Plasma Concentration

**Figure 1:**
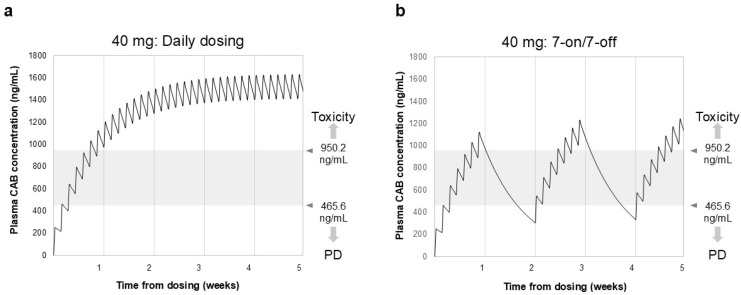
Simulation of blood drug concentration of CAB in daily dosing (**a**) and 7-on/7-off regimen (**b**). C_trough_ represents the minimum plasma concentration measured immediately before each administration of CAB. Gray shading indicates drug efficacy and safety target C_trough_ values. In the daily dosing group, C_trough_ reached 977 ng/mL after 1 week and reached a steady state after 3–4 weeks. In contrast, in the 7-on/7-off regimen, C_trough_ values remained in the safe range of 470–950 ng/mL. CAB, cabozantinib; C_trough,_ trough concentration; PD, progressive disease.

Figure 1 displays the simulation results of blood drug concentrations following daily dosing and the 7-on/7-off regimen of CAB. The trough level reached 977 ng/mL roughly one week after the initiation of daily dosing of CAB (40 mg). In contrast, during the withdrawal period of the 7-on/7-off regimen, the blood concentration of CAB decreased slowly, with most C_trough_ values remaining between 470 and 950 ng/mL.

### 3.3. Therapeutic Efficacy

**Figure 2:**
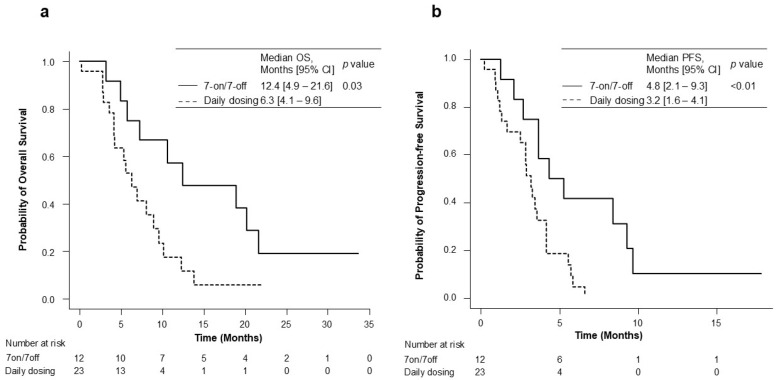
Kaplan–Meier analysis for OS (**a**) and PFS (**b**) in the 7-on/7-off and daily dosing groups. CI, confidence interval; OS, overall survival; PFS, progression-free survival.

The OS and PFS for the 7-on/7-off and daily dosing groups are shown in Figure 2. During the observation period of a median of 6.4 months (range: 4.2–10.5), the median overall survival (OS) in the 7-on/7-off and in the daily dosing groups was 12.4 months (95% CI: 4.9–21.6) and 6.3 months (95% CI: 4.1–9.6). The OS was significantly different between the two groups (*p* = 0.03). The median PFS was also significantly longer in the 7-on/7-off group than in the daily dosing group (4.8 months [95% CI: 2.1–9.3] vs. 3.2 months [95% CI: 1.6–4.1]; *p* < 0.01). The initial therapeutic efficacy at eight weeks is shown in Table 2. ORR, as assessed using RECIST ver. 1.1, was significantly higher in the 7-on/7-off group than that in the daily dosing group (16.7% vs. 0.0%; *p* = 0.04).

### 3.4. Therapeutic Details

**Figure 3:**
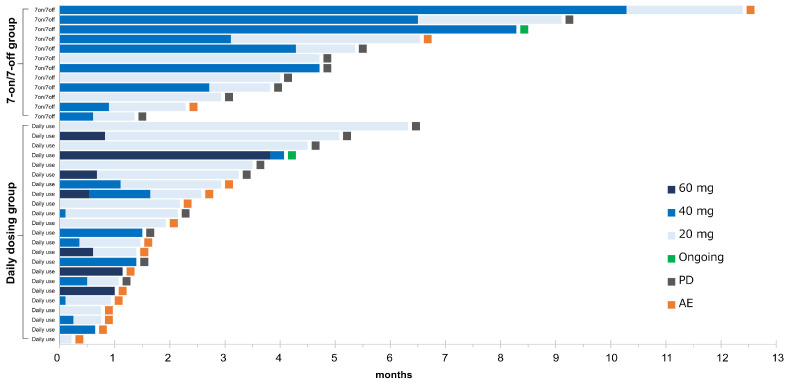
Swimmer plot of all patients. The duration of cabozantinib in each patient is represented by a bar, along with the daily doses, sorted by the duration of treatment in each group. AE, adverse event; PD, progressive disease.

The details in the treatment for the 7-on/7-off and the daily dosing groups are shown in Table 3 and Figure 3. The initial dose of CAB was similar in both groups (*p* = 0.08). The median duration of drug exposure (122 days vs. 42 days) and the median duration of dose reduction (100 days vs. 23 days) were significantly longer in the 7-on/7-off group (*p* < 0.01). In contrast, the median RDI was significantly lower in the 7-on/7-off group than in the daily group (0.27 vs. 0.44, *p* < 0.01). There were no significant differences in dose reduction due to AEs, discontinuation rates, or rates of transition to post-treatment following CAB.

### 3.5. Adverse Events

The frequencies of overall AEs in the two groups are presented in Table 4. A total of 32 of 35 subjects (91.4%) experienced at least one AE of any grade, with grade 3 or 4 AEs reported in 10 of 35 (28.6%) patients. The incidence of AEs of any grade was significantly lower in the 7-on/7-off group than in the daily dosing group (75.0% vs. 100.0%; *p* = 0.03). The incidence of grade 3 or higher AEs tended to be lower in the 7-on/7-off group (16.7% vs. 34.8%, *p* = 0.43). The common AEs of any grade were anorexia (34.3%), fatigue (31.4%), and proteinuria (20.0%). Similarly, commonly noticed grade 3 AEs were anorexia (11.4%), fatigue (8.6%), proteinuria (5.7%), and elevated transaminase levels. The incidence of grade 4 AEs was 2.9% (1/35), with one patient developing pneumonia. No grade 5 AEs were reported.

**Figure 4:**
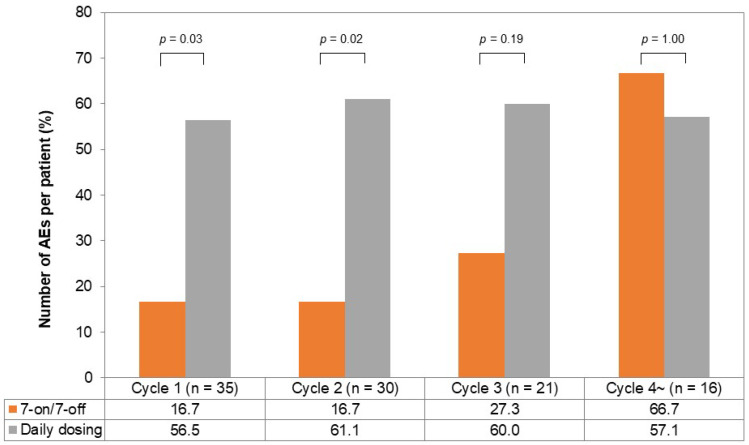
The incidence of AEs per chemotherapy cycle in the 7-on/7-off and daily dosing groups. One chemotherapy cycle was defined as 28 days of cabozantinib administration. The incidence of AEs in cycle one and cycle two was significantly lower in the 7-on/7-off group in comparison to the daily dosing group. AEs, adverse events.

The incidence of AEs per chemotherapy cycle is illustrated in Figure 4. In the first and second cycles, the incidence of AEs was lower in the 7-on/7-off group (cycle one; 16.7% vs. 56.5%, *p* = 0.03, cycle two; 16.7% vs. 61.1%, *p* = 0.02). The incidence of AEs in the third cycle tended to be lower in the 7-on/7-off group (27.3% vs. 60.0%, *p* = 0.19), while it was comparable in the fourth cycle (66.7% vs. 57.1%, *p* = 1.00).

### 3.6. Effect on the Hepatic Functional Reserve

**Figure 5:**
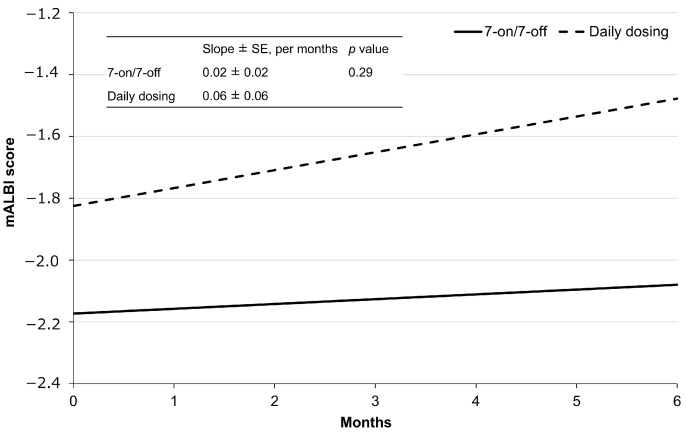
Regression lines by administration method, as determined using mixed-effects models. mALBI, modified albumin-bilirubin; SE, standard error.

The regression curves for mALBI for each group are shown in Figure 5. The mean mALBI before initiating CAB treatment in the 7-on/7-off group was −2.17 ± 0.14 and increased by an average of 0.02 ± 0.02 per month thereafter. In contrast, the mean mALBI prior to the initiation of CAB treatment in the daily dosing group was −1.82 ± 0.29, with a subsequent mean increase of 0.06 ± 0.06 per month. Although the slope between the two groups was not significantly different (*p* = 0.29), the 7-on/7-off group exhibited a trend toward a smaller rate of change in mALBI.

## 4. Discussion

In this study, it was found the 7-on/7-off regimen of CAB for u-HCC could lessen the incidence of AEs and extend the time until drug dose reduction, resulting in extension in the dosing of CAB and survivals.

Previous real-world clinical studies of CAB in Japan reported a median OS of 6.8 to 11.6 months and a median PFS of 2.1 to 5.6 months in u-HCC [6,7,8,26]. The OS and PFS of our patients on the 7-on/7-off regimen were 12.4 and 4.8 months, respectively, the values of which tended to be higher in comparison to previous reports. Also, the 7-on/7-off regimen was more effective than the daily dosing regimen in terms of OS, PFS, and ORR. These results may be explained by the prolonged period until drug dose reduction and prolonged duration of drug exposure in the 7-on/7-off group. Alternatively, the RDI was significantly lower in the 7-on/7-off group than in the daily group. Since Okubo et al. failed to show a close association of PFS and RDI at 6 weeks [8], the long-term administration of CAB seems to have been critical in better survivals in our 7-on/7-off group, regardless of RDI.

Patients with u-HCC tend to have an impaired liver function and worse prognosis under serious AEs in late-line chemotherapy regimens such as CAB. In fact, patients experience reduced therapeutic efficacy and high discontinuation rates owing to AEs during MTA administration [27,28]. Therefore, it is crucial to minimize dose reduction or discontinuation due to AEs during u-HCC treatment. In our study, more than half (57.1%) of the patients received CAB as tertiary or late-line therapy. In this context, it is notable that the 7-on/7-off group experienced fewer AEs of any grade than the daily group, particularly during the initial phase of treatment. In addition, the time to drug dose reduction due to AEs was longer in the 7-on/7-off group than in the daily dosing group.

The blood concentration of CAB administered daily at a dose of 40 mg/day reached 977 ng/dL within one week, and it even increased at a higher concentration thereafter. In contrast, the blood concentration with the 7-on/7-off regimen remained within the C_trough_ value range of 470–950 ng/mL. CAB has a long half-life for approximately 120 h and is highly accumulative, with a fivefold increase in the area on day 15 following a daily dose of 60 mg [29]. It thus can be presumed that AEs occur during the early phase of drug administration. However, the 7-on/7-off regimen can offer a temporary withdrawal period before CAB toxicity accumulates and, as a consequence, fewer AEs, particularly during the early dosing period.

The ALBI score is commonly used as a measure of liver function in patients with HCC, with a decrease in the score indicating poor survival and serious toxicity outcomes [30]. Patients treated with TKIs have also been shown to manifest lower ALBI scores because of tumor progression [31]. Therefore, it is crucial to treat patients with a substantial value of ALBI by CAB. To date, however, no reports have examined changes in mALBI scores under the treatment by CAB. In our present study, a mixed-effects regression model was employed to compare the change in mALBI scores before and after CAB treatment between the 7-on/7-off and the daily dosing groups. While the results indicated no obvious differences in the rate of change in mALBI, there was a trend towards a smaller rate of the change in the 7-on/7-off group. It was thus suggested that the 7-on/7-off regimen may contribute to the lower decline in the hepatic reserve capacity in patients with u-HCC. This speculation awaits further elucidations.

This study was associated with several limitations. First, while this was a multicenter study, the number of patients was relatively small. This may have contributed to biases in the tumor and patient characteristics. A larger prospective and randomized clinical trial is needed to further demonstrate the usefulness and tolerability of the 7-on/7-off regimen. Second, we could not measure the actual patient blood levels of CAB in the current study. This seems to have contributed to the obscurity in the clearance and exposure of CAB among individuals [32]. Third, systemic therapy for unresectable hepatocellular carcinoma primarily consists of immunotherapy, with CAB treatment being utilized in a more limited patient population. While we acknowledge the value of investigating the associations between laboratory parameters, tumor characteristics, and treatment outcomes through a multivariate analysis, our relatively small sample size precluded such a comprehensive statistical evaluation. Fourth, we were only able to study the 40 mg dose and could not simulate and compare the concentrations of 60 mg and 20 mg of CAB, which would have provided valuable information on dose–response relationships and optimal dosage. However, we believe that the efficacy and safety of the 7-on/7-off regimen have been demonstrated in patients with u-HCC.

## 5. Conclusions

The 7-on/7-off regimen of CAB for u-HCC was shown to have potential to improve the tolerability and treatment response in conventional CAB treatment. In particular, the protocol was shown to play an important role in reducing the incidence of AEs in the early phase of dosing, thereby making long-term dosing possible. Further studies are required to validate our results.

## Figures and Tables

**Table 1:** Baseline characteristics.

Variable	Total (*n* = 35)		7-on/7-off (*n* = 12)		Daily Dosing (*n* = 23)		*p* Value
Age, years	67.2	(63.8–75.0)	74.8	(70.0–82.4)	66.6	(62.8.0–72.5)	0.07
Male sex	32	(91.4)	10	(83.3)	22	(95.7)	0.27
BMI, kg/m^2^	23.3	(20.2–26.0)	22.7	(20.7–25.4)	23.8	(20.0–26.5)	0.61
Etiology, HBV/HCV/Non-Viral	5/15/15		2/3/7		3/12/8		0.29
ECOG PS, 0	31	(88.6)	10	(83.3)	21	(91.3)	0.48
Treatment line 2nd/3rd/4th/5th	15/12/7/1		7/3/2/0		8/9/5/1		0.55
Prior treatment, LEN/Atez + Bev or Dur + Tre	15/20		7/5		8/15		0.18
mALBI grade 1/2a/2b/3	15/12/7/1		7/3/2/0		8/9/5/1		0.55
BCLC stage C	28	(80.0)	9	(75.0)	19	(82.6)	0.67
Tumor size, cm	6.9	(3.5–8.6)	7.6	(5.8–13.1)	7.4	(6.0–9.7)	0.57
Tumor number, multiple	29	(82.9)	9	(75.0)	20	(87.0)	0.39
Macrovascular invasion	13	(37.1)	6	(50.0)	7	(30.4)	0.29
Extrahepatic spread	18	(51.4)	5	(41.7)	13	(56.5)	0.49
AFP, ≥400 ng/mL	12	(34.3)	3	(25.0)	9	(39.1)	0.48
DCP, ≥400 mAU/mL	24	(68.6)	5	(50.0)	18	(78.3)	0.13

Data are presented as the median (interquartile range) or n (%). AFP, alpha-fetoprotein; Atez + Bev, atezolizumab plus bevacizumab; BCLC, Barcelona Clinic Liver Cancer; DCP, des-γ-carboxy prothrombin; Dur + Tre, Durvalumab plus tremelimumab; ECOG PS, Eastern Cooperative Oncology Group performance status; LEN, Lenvatinib; mALBI, modified albumin-bilirubin.

**Table 2:** Initial assessment of the therapeutic response according to RECIST ver. 1.1 and mRECIST.

	RECIST ver. 1.1							mRECIST						
	Total (*n* = 35)		7-on/7-off (*n* = 12)		Daily Dosing (*n* = 23)		*p* Value	Total (*n* = 35)		7-on/7-off (*n* = 12)		Daily Dosing (*n* = 23)		*p* Value
CR	0	(0.0)	0	(0.0)	0	(0.0)		0	(0.0)	0	(0.0)	0	(0.0)	
PR	2	(5.7)	2	(16.7)	0	(0.0)		7	(20.0)	4	(33.3)	3	(13.0)	
SD	19	(54.2)	7	(58.3)	12	(52.2)		14	(40.0)	5	(41.7)	9	(39.2)	
PD	11	(31.4)	3	(25.0)	8	(34.8)		11	(31.4)	3	(25.0)	8	(34.8)	
NE	3	(8.6)	0	(0.0)	3	(13.00)		3	(8.6)	0	(0.0)	3	(13.00)	
ORR	5.7%		16.7%		0.0%		0.04 *	20.0%		33.3%		13.0%		0.16
DCR	59.9%		75.0%		52.2%		0.19	60.0%		75.0%		52.2%		0.19

Data are presented as n (%). CR, complete response; DCR, disease control rate; mRECIST, modified RECIST; NE, not estimable; ORR, objective response rate; PD, progressive disease; PR, partial response; RECIST, Response Evaluation Criteria in Solid Tumors; SD, stable disease. * Significant factors with *p*-values < 0.05.

**Table 3:** Therapeutic details.

	Total (*n* = 35)	7-on/7-off (*n* = 12)	Daily Dosing (*n* = 23)	*p* Value
Initial dose of cabozantinib, 20 mg/40 mg/60 mg, n	10/19/6	3/9/0	7/10/6	0.08
Median duration of drug exposure, days (range)	72 (39–120)	122 (90–192)	42 (29–87)	<0.01 *
Median relative dose intensity, % (range)	0.33 (0.30–0.48)	0.27 (0.22–0.30)	0.44 (0.33–0.65)	<0.01 *
Median duration of dose reduction, days (range)	39 (18–103)	100 (73–145)	23 (15–48)	<0.01 *
AE-related dose reductions, n (%)	18 (51.4)	7 (58.3)	11 (47.8)	0.73
AE-related dose discontinuation, n (%)	17 (48.6)	4 (33.3)	13 (56.5)	0.29
Reason for discontinuation, AE/PD, n	18/15	5/6	13/9	0.54
Transition to posttreatment after cabozantinib, n (%)	8 (24.2)	4 (36.4)	4 (18.2)	0.39
Outcome, survival, n (%)	12 (34.3)	7 (58.3)	5 (21.7)	0.06
Observation period, months	6.4 (4.2–11.6)	11.5 (6.9–20.8)	5.4 (3.8–8.6)	0.01 *

AE, adverse event; PD, progressive disease. * Significant factors with *p*-values < 0.05.

**Table 4:** Summary of treatment-emergent adverse events.

	Any Grade				Grade ≥ 3			
	Total (*n* = 35)	7-on/7-off (*n* = 12)	Daily Dosing (*n* = 23)	*p* Value	Total (*n* = 35)	7-on/7-off (*n* = 12)	Daily Dosing (*n* = 23)	*p* Value
Any cause	32 (91.4)	9 (75.0)	23 (100.0)	0.03 *	10 (28.6)	2 (16.7)	8 (34.8)	0.43
Anorexia	12 (34.3)	4 (33.3)	8 (34.8)	1.00	4 (11.4)	1 (8.3)	3 (13.0)	1.00
Fatigue	11 (31.4)	4 (33.3)	7 (30.4)	1.00	3 (8.6)	1 (8.3)	2 (8.3)	1.00
Proteinuria	7 (20.0)	2 (16.7)	5 (21.7)	1.00	2 (5.7)	1 (8.3)	1 (4.3)	1.00
Diarrhea or colitis	5 (14.3)	0	5 (21.7)	0.14	0	0	0	
Edema	4 (11.4)	1 (8.3)	3 (13.0)	1.00	0	0	0	
Increased transaminase	3 (8.6)	1 (8.3)	2 (8.7)	1.00	2 (5.7)	0	2 (8.7)	0.54
Pruritus or rash	3 (8.6)	2 (16.7)	1 (4.3)	0.27	0	0	0	
Palmar-plantar erythrodysesthesia syndrome	2 (5.7)	0	2 (8.7)	0.54	1 (2.9)	0	1 (4.3)	1.00
Hypertension	2 (5.7)	0	2 (8.7)	0.54	0	0	0	
Pneumonia	1 (2.9)	0	1 (4.3)	1.00	1 (2.9)	0	1 (4.3)	1.00
Adrenal insufficiency	1 (2.9)	0	1 (4.3)	1.00	0	0	0	
Pyrexia	1 (2.9)	0	1 (4.3)	1.00	0	0	0	

Data are presented as n (%). * Significant factors with *p*-values < 0.05.

## Data Availability

The data presented in this study are available upon request from the corresponding author.
